# Hybrid system for rechargeable magnesium battery with high energy density

**DOI:** 10.1038/srep11931

**Published:** 2015-07-15

**Authors:** Zheng Chang, Yaqiong Yang, Xiaowei Wang, Minxia Li, Zhengwen Fu, Yuping Wu, Rudolf Holze

**Affiliations:** 1 New Energy and Materials Laboratory (NEML), Department of Chemistry & Shanghai Key Laboratory of Molecular Catalysis and Innovative Material, Fudan University, Shanghai 200433, China; 2 College of Energy, Nanjing Tech University, Nanjing 211816, Jiangsu Province, China; 3 Technische Universität Chemnitz, Institut für Chemie, AG Elektrochemie, Chemnitz D-09107, Germany.

## Abstract

One of the main challenges of electrical energy storage (EES) is the development of environmentally friendly battery systems with high safety and high energy density. Rechargeable Mg batteries have been long considered as one highly promising system due to the use of low cost and dendrite-free magnesium metal. The bottleneck for traditional Mg batteries is to achieve high energy density since their output voltage is below 2.0 V. Here, we report a magnesium battery using Mg in Grignard reagent-based electrolyte as the negative electrode, a lithium intercalation compound in aqueous solution as the positive electrode, and a solid electrolyte as a separator. Its average discharge voltage is 2.1 V with stable discharge platform and good cycling life. The calculated energy density based on the two electrodes is high. These findings open another door to rechargeable magnesium batteries.

Without a doubt, electrical energy storage (EES) system of environmentally friendly, high safety and high energy density is highly demanded^1^,^2^,^3^. Although lithium ion batteries (LIBs) show good promise with some quite dominant advantages over conventional batteries, inherent safety issue related to dendrite formation on the negative electrode and high cost still prevent them from demonstrating for large-scale energy storage^4^,^5^. Li//S and Li//air batteries have very high theoretical energy density, but their reversibility, efficiency and cycling are still faced with some challenges^6^,^7^,^8^,^9^.

The relatively higher volumetric capacity (3833 mAh cm^–3^, and 2061 mAh cm^–3^ for Li metal) and lower cost (about 5% of Li metal) of magnesium make it as an excellent candidate for negative electrode material of batteries^10^,^11^,^12^. What’s more, magnesium (Mg) metal cannot be plagued by dendrite formation and is much safer than Li when exposed to air or during fast charging process. Rechargeable magnesium batteries (RMBs) were invented in the early 2000s^13^,^14^,^15^. Nevertheless, some fatal problems such as the lack of suitable positive electrode materials and electrolytes of wide electrochemical windows remain unsolved^16^,^17^,^18^,^19^,^20^,^21^,^22^,^23^. So far, reversible Mg deposition and dissolution are mostly realized in ethereal solutions of Grignard reagents RMgX (where R = alkyl, or aryl groups, and X = Cl or Br)^16^. This electrolyte has low anodic stability and narrow electrochemical window though some improvements have been achieved^17^. Some efforts were aimed to develop high voltage electrolytes such as magnesium organohaloaluminates, ion liquid electrolyte, and boron-based electrolytes^18^,^19^. However, the RMBs deliver rather lower average output voltage (below 2.0 V)^20^,^21^,^22^. In addition, the kinetically sluggish diffusion for Mg^2+^ cation in the positive electrode materials limits its power and energy densities, and there are only a few matrixes for electrochemically reversible intercalation compounds for magnesium such as Chevrel phase Mo_6_S_8_, nano-crystalline V_2_O_5_ and magnesiation of olivines^23^,^24^,^25^. As a result, the energy density of Mg-based rechargeable battery is rather low due to the small capacity of the positive electrodes and low electrochemical windows of the electrolytes. Recently, a conversion-type positive electrode based on S with an extremely large capacity was found^11^. However, its Coulomb efficiency and discharge voltage are still low. To achieve high energy density, the average output voltage is of paramount importance. Former hybrid magnesium and lithium batteries tried to combine the advantages of Li and Mg electrochemistry, but their discharge plateaus (1.66 V and 1.29 V) are much lower compared with lithium batteries^22^.

Here we report a rechargeable magnesium battery which consists of a lithium intercalation compound in aqueous electrolyte instead of Mg ions insertion compounds as the positive electrode, Mg in nonaqueous electrolyte based on Grignard reagent as the negative electrode, and Cu as the current collector of the negative electrode for deposition and dissolution of magnesium. Its average discharge voltage can be up to 2.1 V with stable discharge platform and good cycling, and its energy density can be comparable with those of the corresponding lithium ion batteries.

## Results

### Redox reactions of Mg and LiFePO_4_ electrodes

**Figure 1:**
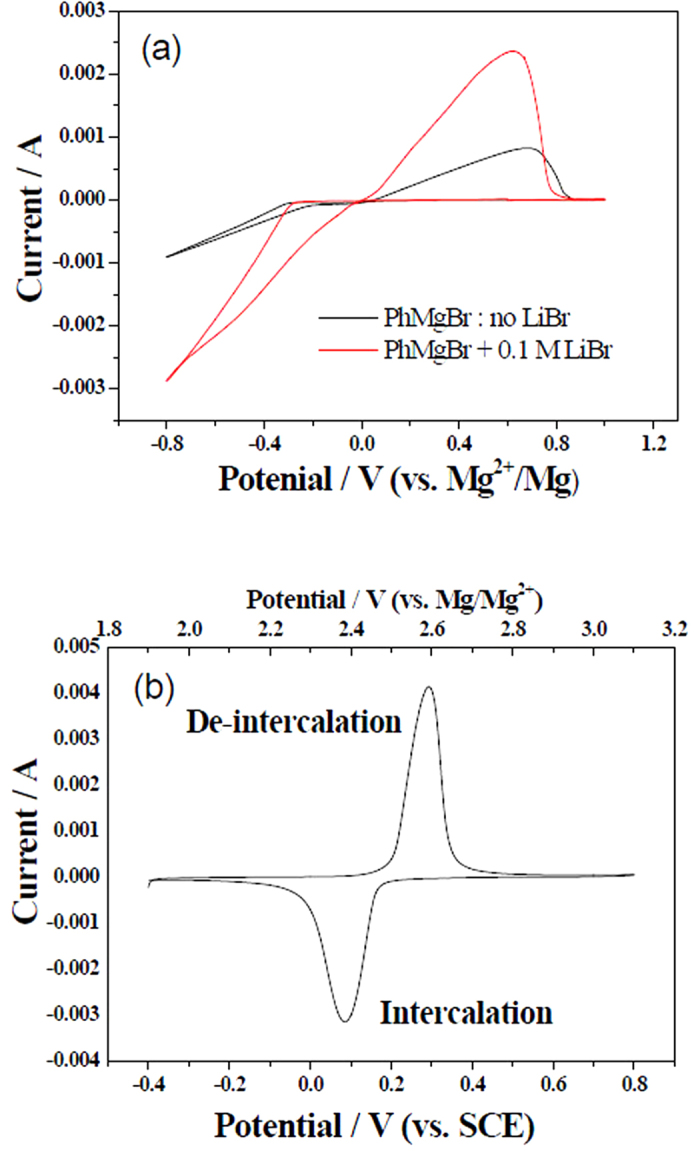
Cyclic voltammograms (CVs) measured at the scan rate of 1 mV s^−1^ for (**a**) Mg in tetrahydrofuran (THF) solution containing 1 M PhMgBr and 0.1 M LiBr and (**b**) LiFePO_4_ in 0.5 M Li_2_SO_4_ aqueous solution by using Ni mesh and saturated calomel electrode (SCE) as the counter and the reference electrodes, respectively.

Cyclic voltammograms (CVs) for the copper in PhMgBr/THF solution with and without addition of LiBr and LiFePO_4_ in the aqueous electrolyte is shown in Fig. 1(a). The reversible magnesium plating (deposition)/dissolution process is very clear. In the PhMgBr/THF electrolyte without LiBr, magnesium plating commences at 0.0 V (vs. Mg^2+^/Mg). After LiBr (0.1 M) was added, the plating current density increases. The potential for the dissolution of Mg also decreases from 0.69 to 0.60 V (vs. Mg^2+^/Mg). It is interesting to find that the added LiBr improves the kinetics of the Mg plating/dissolution processes. As to the detailed mechanism, perhaps it is due to the higher ionic conductivity from the added LiBr^21^. However, the lithium salt is necessary to balance charge as illustrated in the following. Additionally, Li ions are not reduced and deposited on the surface of the Cu substrate because the deposition potential of Li^+^ ions is much lower than that of Mg^2+^. Of course, other treated metals such as Ca and Al can also be used as the negative electrode material^26^.

**Figure 2:**
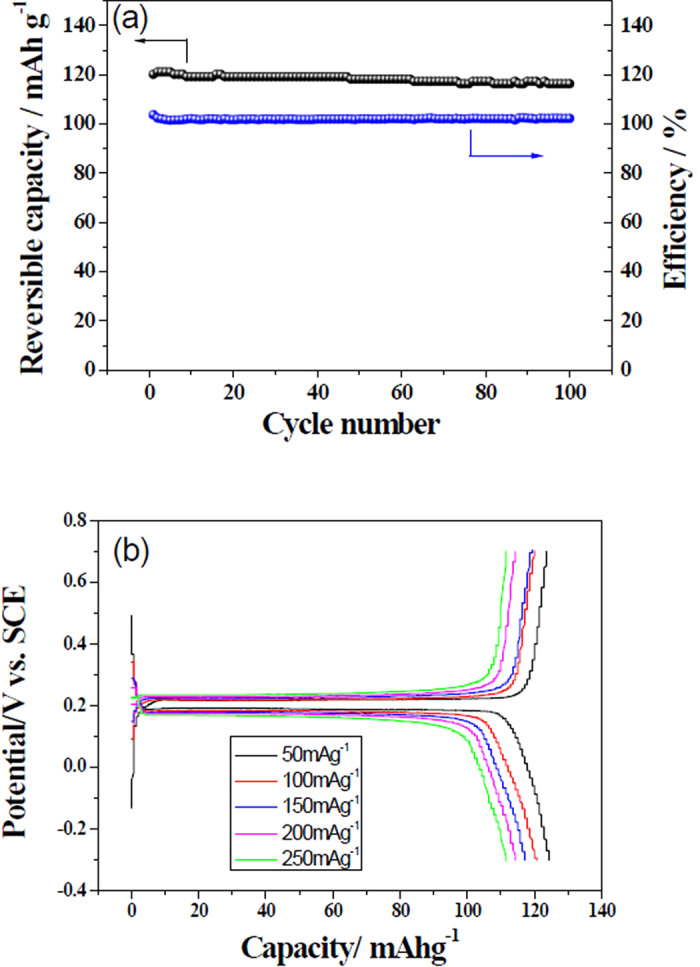
Cycling performance at the current density of 50 mA g^−1^ (**a**) and the charge–discharge curves at the different current density (**b**) of LiFePO_4_ positive electrode in 0.5 M Li_2_SO_4_ aqueous electrolyte, which was tested by using Ni mesh at the counter electrode.

In the case of our prepared three dimensional porous LiFePO_4_ (see Figure S1 for morphology and X-ray diffraction in ESI: electronic supporting information), its main redox peaks for the de-intercalation/intercalation of Li^+^ ions in 0.5 M Li_2_SO_4_ aqueous solution are located at 0.3 V and 0.1 V (vs. SCE) (Fig. 1b), respectively, which are consistent with the formerly reported intercalation/deintercalation behavior of LiFePO_4_ in the aqueous electrolytes^27^,^28^. Its cycling performance in the aqueous electrolyte is shown in Fig. 2. It is clear that there is no evident capacity fading with initial capacity of about 120 mAh/g and its coulomb efficiency is 100%^28^. At a charge-discharge current density of 50, 150, 200, and 250 mA g^−1^, its capacity is 124, 120, 117, 114, and 111 mA h g^−1^, respectively (Fig. 2b), which is evidently superior to the reported behavior for LiFePO_4_ in organic electrolytes. The macroporous morphology and preferred degree of crystallinity of LiFePO_4_ are the main reasons for its excellent electrochemical performance^28^.

### Electrochemical performance of the assembled Mg//LiFePO_4_ battery

**Figure 3:**
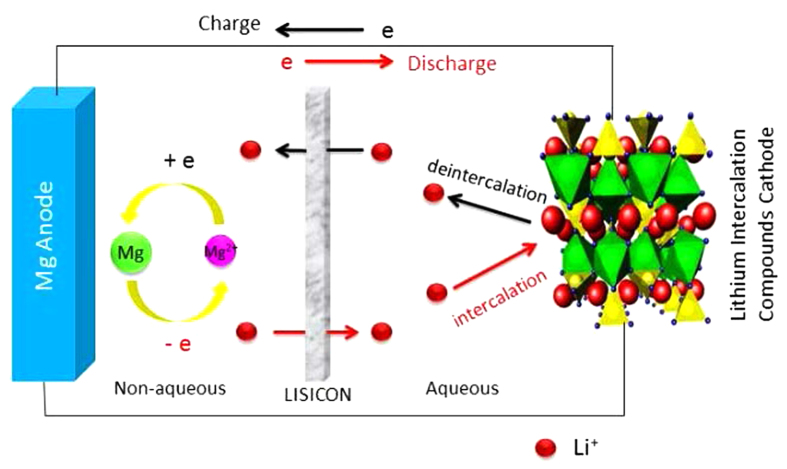
Schematic illustration of our designed rechargeable magnesium battery using the magnesium in PhMgBr-based organic electrolyte with a small quantity of LiBr as a negative electrode, LiFePO_4_ in 0.5 M Li_2_SO_4_ aqueous electrolyte as a positive electrode, and LISICON solid electrolyte as a separator.

The above results show clearly that both Mg and LiFePO_4_ can take place reversible redox reactions. The standard potential of Mg^2+^/Mg is –2.36 V (vs. NHE, normal hydrogen electrode), and that for FePO_4_/ LiFePO_4_ is 0.42 V (vs. NHE). As a result, their combination will build up a rechargeable battery system with a high output voltage of about 2.78 V. However, when traditional porous separators are used to separate the negative and positive electrodes, the organic electrolyte will pass through the pores and mix with the aqueous electrolyte, leading to poor stability. It was reported that a solid electrolyte can separate an organic electrolyte with an aqueous one and only Li^+^ ions can be reversibly transported^28^,^29^,^30^,^31^,^32^. Consequently, a LISICON film consisting of Li_2_O-Al_2_O_3_-50SiO_2_-P_2_O_5_ -TiO_2_-GeO_2_ (Ohara Inc., Japan) was used as a separator to separate the aqueous and nonaqueous electrolytes, whose thickness and lithium ionic conductivity are 100 μm and 0.1 mS cm^−1^ at room temperature, respectively. The schematic structure of the assembled hybrid Mg battery is shown in Fig. 3.

**Figure 4:**
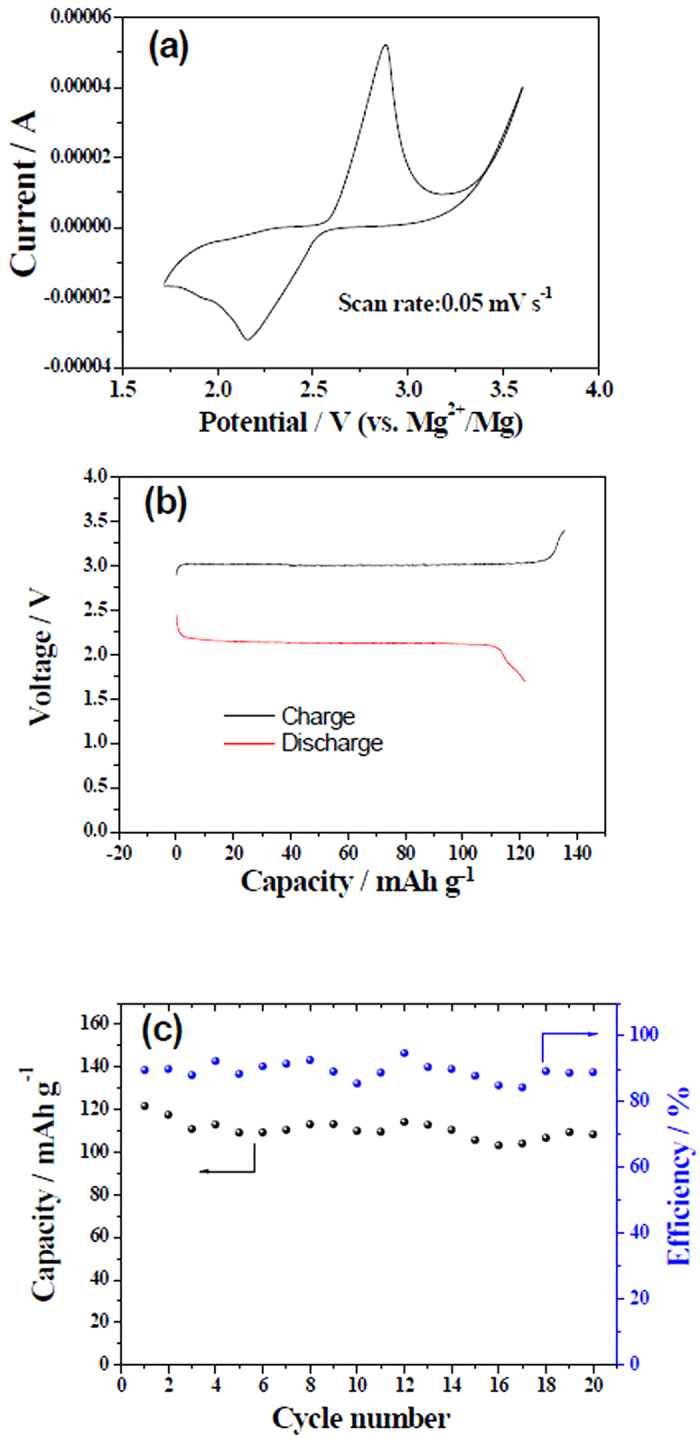
Electrochemical performance of the assembled rechargeable magnesium battery: (**a**) CV curve at the scan rate of 0.05 mV s^−1^, (**b**) galvanostatic charge-discharge curves in the first cycle at the current density of 50 mA g^−1^ based on the positive electrode between 1.7 and 3.4 V, and (**c**) the cycling performance between 1.7 and 3.4 V at the current density of 50 mA g^−1^ based on the LiFePO_4_ positive electrode.

Some primary electrochemical performance of the assembled battery is shown in Fig. 4. In the CV curve at the scan rate of 0.05 mV s^−1^ there is a couple of redox peaks situated at 2.88 and 2.16 V (Fig. 4a), respectively. This means that the battery can normally operate. During the charging process, at the positive electrode side Li^+^ ions de-intercalate from LiFePO_4_ by giving electrons and go into the aqueous solution, pass through the LISICON film, and then enter into the non-aqueous electrolyte to keep charge balance or neutrality. At the negative electrode side, Mg^2+^ ions in the Grignard reagent move to the surface of the Cu foil and plate as metallic Mg by getting 2 electrons for each Mg^2+^ ions. During the discharging process, the reverse processes take place. The plated Mg dissolves into the non-aqueous electrolyte at the negative electrode side by giving electrons. At same time, Li^+^ ions in the non-aqueous electrolyte pass across LISICON film to aqueous electrolyte solution to keep charge balance in the electrodes and the electrolytes, and then intercalate into the LiFePO_4_ positive electrode reversely by getting electrons. It is worthy to mention that when one Li^+^ ion passes from aqueous side to the non-aqueous side during the charging process, then one Li^+^ ion comes back to the aqueous side from non-aqueous side during the discharging process. After one full cycle the concentrations of Li^+^ ions in the electrolytes of both sides are recovered. It should also be noted that only lithium ions can pass across the LISICON film during the charge/discharge processes though the size of Mg^2+^ (0.072 nm) is similar to that of Li^+^ (0.076 nm). However, since its charge density is too large, it could not enter into the lattice structure to replace Li^+^ ions, which is similar to the difficult intercalation of Zn^2+^ into LiMn_2_O_4_ though the radius of Zn^2+^ (0.074 nm) is also smaller than that of Li^+^^32^. Consequently, the electrode reactions are simply shown as the following:

Positive electrode reaction:


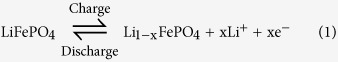


Negative electrode reaction (Note: The real reactions are complicated due to the existence of Mg complexes):


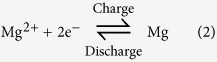


Total reaction:


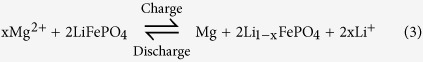


In the first cycle between 1.7 and 3.4 V at 50 mA g^−1^ based on the positive electrode (Fig. 4b), there are two distinct voltage plateaus, namely a charge plateau at 3.02 V and a discharge one at 2.12 V, which are consistent with the above CV results. This is also in good agreement with the above mentioned charge and discharge processes. The average discharge voltages are about 2.1 V, higher than those of the reported Mg batteries based on the magnesium intercalation positive electrodes, below 1.8 V^19^. The good plateau with high voltage is a great improvement compared with the former prototype of a high energy-density rechargeable Mg battery^20^. However, there is a big polarization between the charge and discharge processes. Perhaps it is due to the ohmic polarization of LISICON as mentioned before since its lithium ionic conductivity is only about 0.1 mS cm^−1^. Another reason is due to the slow plating and dissolution processes of magnesium and the low ionic conductivity of the Grignard reagent. At 50 mA g^−1^ the initial charge and discharge capacities of this battery based on the mass of LiFePO_4_ are 135.8 and 121.7 mAh g^−1^ (Fig. 4b), respectively, and the initial coulomb efficiency is 89.7%. Its reversible capacity is similar to that of the LiFePO_4_ positive electrode in the aqueous electrolytes. The assembled magnesium battery presents satisfactory capacity retention, with 10% capacity loss after 20 full cycles at the current density of 50 mA g^−1^ based on the mass of LiFePO_4_ (Fig. 4c), superior to the recent reported dual-salt polyvalent-metal storage battery^26^. The capacity and cycling stability are out of our expectation since the Grignard reagent is very sensitive with water and the absorbed small amount of water on the surface of LISICON film affects the plating/dissolution of magnesium. During the cycling at 50 mA g^−1^ the Coulomb efficiency is less than 100% because of the low efficiency for the magnesium plating/dissolution in the Grignard reagent (see Figure S2 in ESI).

Based on the average discharge voltages and the capacities of the magnesium negative electrode and the LiFePO_4_ positive electrode, the discharge energy density of this battery based on the total mass of the electrode materials is 245 Wh kg^−1^ (see ESI for the calculation), which is comparable with that of lithium ion batteries based on graphite//LiFePO_4_.

## Discussion

As well-known, Mg is highly reactive with water, O_2_ and CO_2_, which results in insulating MgO layers, thus inhibiting further reactions. It is impossible to use aqueous solution for rechargeable Mg battery. In addition, the potential of magnesium metal is much lower than that for hydrogen evolution. Furthermore, Grignard reagent can react with H_2_O violently, and its electrochemical window is very narrow (1.5 V) (see Figure S3 in ESI). However, in our case, Mg metal is very stable in the Grignard reagent of PhMgBr, whose cycling life can be above 1000 since there is no dendrite^16^,^18^,^21^. In addition, it does not contact the aqueous electrolyte in the positive electrode side, and there is no hydrogen evolution. The main reason is that Li^+^ ions acting as the charge transfer media which can cross over the hydrogen evolution potential range through LISICON and arrive at the magnesium metal directly^29^,^30^. This cross-over is similar to the potential change between both sides of a cell membrane, and the potential of Li^+^ ions decreases very sharply from the positive electrode to the negative one (see Figure S4 in ESI). The Li^+^ ions in the positive electrode side have higher potential and are very stable. Meanwhile, water and protons could not enter into the negative side, so they could not arrive at enough low potential to produce hydrogen. As to the LiFePO_4_ positive electrode, it is stable in water since its potential is below that for the oxygen evolution and much higher than that for hydrogen evolution^27^,^28^. LISICON film prevents the contact of the Grignard reagent with the aqueous electrolyte so that the applied voltage is not high enough for the Grignard reagent to decompose. As a result, this hybrid system solves the possible oxidation or decomposition of the Grignard reagent-based electrolytes at the positive electrode and avoids the reduction of protons or water at the negative electrode, and the charge and discharge voltages are very stable even after 20 cycles (see Figure S5 in ESI).

Compared with the recent reported dual-salt polyvalent-metal storage battery^26^, our cycling is much better since the ether-based electrolyte can still decompose though AlCl_3_ is added to stabilize the anions. In addition, Mg^2+^ can intercalate into FePO_4_ structure, leading to structure fading of LiFePO_4_^33^.

At present, there are some problems related to the possible practical applications by adopting the solid state electrolytes (LISICON) due to the following two reasons: (1) Its cost is high, and future methods to decrease its manufacturing cost are needed since its primary materials are not expensive; and (2) its ionic conductivity at room temperature is not high enough and a large overpotentials or polarizations are produced. It is delighting that many endeavours are under way^34^. As a result, it will be a promising energy storage system.

If other intercalation compounds such as LiMn_2_O_4_, LiCoO_2_ and Li[Ni_1/3_Co_1/3_Mn_1/3_]O_2_, which are stable in aqueous electrolytes, are used as the positive electrode^35^,^36^,^37^,^38^,^39^,^40^, not only the average discharge voltage will be higher but also the energy density and cycling performance will be improved. For example, its theoretic energy density based on Mg and LiCoO_2_ can be up to 450 Wh kg^−1^ (see ESI for the calculation), which can be comparable with those of lithium ion batteries for electric vehicles. In practical battery the energy density will be only about half due to the need of other components such as electrolytes, separators, current collectors and case.

In summary, our above work provides a hybrid system for rechargeable magnesium battery by using Mg in a Grignard reagent-based organic electrolyte to improve their electrochemical stable windows^26^ as the negative electrode, lithium intercalation compound in aqueous solution as the positive electrode, and solid electrolyte as the separator. This system avoids the oxidation of Grignard reagent-based electrolyte, and broadens the average output voltage that is 2.1 V, higher than those of the formerly reported rechargeable magnesium batteries. The used lithium intercalation compounds solve the difficulty of Mg^2+^ intercalation, which is a sluggish process. The energy density based on the two electrodes for this hybrid system can be comparable with those of the corresponding lithium ion batteries. The cycling life is also very good. It provides another direction to rechargeable magnesium batteries.

## Method

### Preparation of LiFePO_4_

The preparation process for the LiFePO_4_ is according to our former work^28^. Polystyrene (PS) particles from an emulsion polymerization are used as a sacrificial template. Fe(NO_3_)_3_·9H_2_O, CH_3_COOLi·2H_2_O and H_3_PO_4_ (85%) in stoichiometric amounts of 1:1:1 were dissolved in deionized water, stirred for 2 h, and soaked into the solid PS templates obtained from the emulsion polymerization. After drying under air flow overnight, the sample was heated to 250 ^o^C at a rate of 2 ^o^C min^−1^, and then kept at this temperature for 3 h to harden the inorganic skeleton. Then, the sample was further heated to 600 ^o^C at the same rate and kept at this temperature for another 3 h under a reducing atmosphere (95% Ar and 5% H_2_) to obtain LiFePO_4_. X-ray powder diffraction (XRD) was carried out using a Bruker Analytical X-ray System with Cu Kα radiation source filtered by a thin nickel plate. Scanning electron micrographs (SEM) were obtained with a Philips XL30 scanning electron microscope.

### Assembling of rechargeable magnesium battery

A non-aqueous electrolyte (1 M PhMgBr and 0.1 M LiBr in tetrahydrofuran) was used for the negative electrode side and an aqueous electrolyte (0.5 M Li_2_SO_4_) for the positive electrode side. A LISICON film with component of Li_2_O-Al_2_O_3_-SiO_2_-P_2_O_5_-TiO_2_-GeO_2_ with thickness of 100 μm and conductivity of 10^–4^ S cm^−1^ (Ohara Inc., Japan) was used as a separator. A copper foil (surface area: 0.25 cm^2^) deposited with some amount of magnesium was used as the negative electrode. The as-prepared LiFePO_4_ was mixed with acetylene black and poly(tetrafluoroethylene) (PTFE) in a weight ratio of 8:1:1 with the help of ethanol. After drying, the mixture was pressed into a film with an active mass loading of 3.75 mg cm^–2^, then the film was cut into disks. These disks were pressed onto Ni-grid at a pressure of 10 MPa and then dried at 80°C for one night to act as the positive electrode.

### Electrochemical testing

Cyclic voltammetry (CV) and galvanostatic charging/discharging were performed at room temperature on an electrochemical working station CHI600C (Chenhua, Shanghai, China) and a cell tester (Land, Wuhan, China), respectively.

## Additional Information

**How to cite this article**: Chang, Z. *et al.* Hybrid system for rechargeable magnesium battery with high energy density. *Sci. Rep.* **5**, 11931; doi: 10.1038/srep11931 (2015).

## Supplementary Material

Supplementary Information
