# Supplementary material for: Hybrid system for rechargeable magnesium battery with high energy density
Source: Sci Rep. 2015 Jul 15;5:11931. doi: 10.1038/srep11931 (PMC4502397; doi:10.1038/srep11931)
Supplement: Supplementary Information [file srep11931-s1.pdf]

Electronic supporting information (ESI):

Hybrid system for rechargeable magnesium battery with high energy density

Zheng Chang, Yaqiong Yang, Xiaowei Wang, Minxia Li, Zhengwen Fu, Yuping Wu*, Rudolf Holze

1. Physical characterization of the prepared $LiFePO_4$.

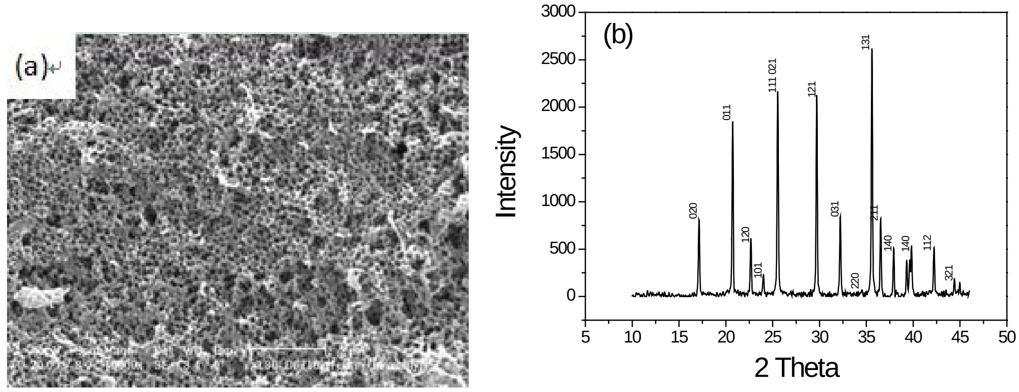

Fig. S1. (a) SEM and (b) X-ray diffraction pattern of $LiFePO_4$.

This 3D macroporous structure will facilitate the penetration of the aqueous electrolyte and give a large accessible surface area to enhance the exchange of Li^+ between theaqueous electrolyte and the electrode. Moreover the XRD pattern of the 3D macroporous $LiFePO_4$ can be well indexed to the pure LiFePO4 phase with an orthorhombic structure (JCPDS card no. 83-2092)

2. Mg deposition/stripping Coulombic efficiency in PhMgBr and PhMgBr+LiBr electrolytes

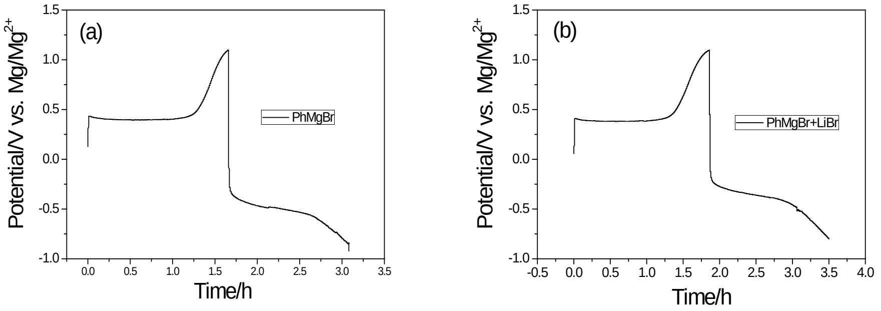

Fig. S2 Typical galvanostatic curves for Mg deposition-dissolution on Cu substrate in 1 M PhMgBr/THF (a), PhMgBr + LiBr/THF (b)

According to the ratio of the charge amount of magnesium dissolution to that of magnesium deposition shown in the above figure, Mg deposition/stripping Coulombic Efficiency in PhMgBr and PhMgBr+LiBr electrolyte are calculated to be 85.5% and 88.2%, respectively.

3. The electrochemical window of the organic electrolyte

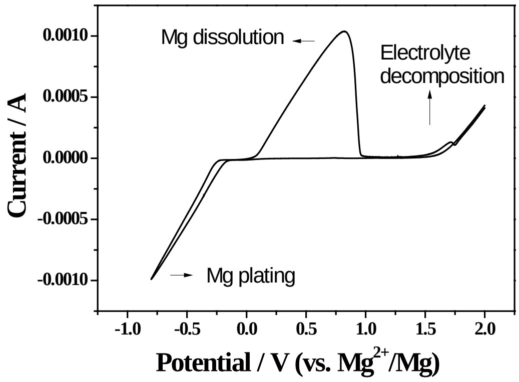

Fig. S3 The typical steady-state voltammograms of Cu electrode in the Grignard reagent PhMgBr of THF solution.

The electrochemical window of this electrolyte is 1.5 V. Above 1.5 V, it begins to decompose.

4. Calculation of the energy density assembled Mg//$LiFePO_4$ battery

Mg: 2200 mAh g^{-1}
$LiFePO_4$: 121.7 mAh g^{-1}
Discharge Potential: 2.12V
The energy density is 121.7 / (1+121.7/2200) x 2.12 = 245 Wh kg^{-1}.

5. The potential change of Li^+ ions in the LISICON film

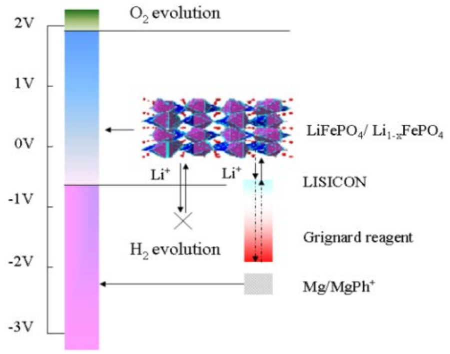

Fig. S4 Schematic illustration of the potential change of Li^+ ions during the movement between the $LiFePO_4$ positive electrode in the aqueous electrolyte and the magnesium in the Grignard reagent-based electrolyte.

6. Charge and discharge curves at different cycles for the rechargeable Mg battery

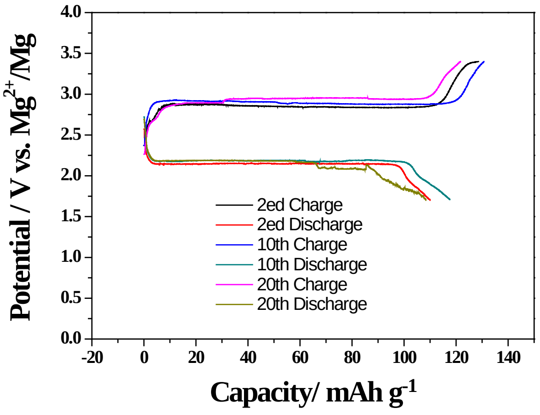

Fig. S5 The charge and discharge curves in the second, tenth, twentieth cycles for the rechargeable Mg//$LiFePO_4$ battery.

7. Calculation of the energy density based on Mg and $LiCoO_2$ on the basis of the theoretic data

Mg: 2200 mAh g^{-1}
$LiCoO_2$: 145 mAh g^{-1}
Potential difference: 0.95 – (-2.37) = 3.32 V
The energy density is 145 / (1+145/2200) x 3.32 = 451 Wh kg^{-1}.
